# The Involvement of the Intestinal Microflora in the Expansion of CD4^+^ T Cells with a-Naive Phenotype in the Periphery

**DOI:** 10.1155/1992/57057

**Published:** 1992

**Authors:** Ruud Dobber, Anita Hertogh-Huijbregts, Jan Rozing, Kim Bottomly, Lex Nagelkerken

**Affiliations:** 1 Section of Immunology Institute of Ageing and Vascular Research TNO P.O. Box 430 Leiden 2300 AK The Netherlands; 2 Section of Immunobiology Howard Hughes Medical Institute Yale University School of Medicine New Haven Connecticut USA

**Keywords:** CD4^+ T cells, germ-free, naive, memory, intestinal microflora

## Abstract

It is well known that immune reactivity declines with age. Recently, we demonstrated that the age-related decrease in IL-2 production by CD4^+^  T cells was accompanied by an increased production of IL-4 and interferon-*γ*,(IFN-*γ*). This age-related shift in the profile of lymphokine production was related to phenotypic changes within the CD4^+^ T-cell subset, that is, a decrease in the percentage of CD45RB^++^ CD4^+^ T cells and an increase in the percentage of Pgp-1^+^ CD4^+^ T cells. To study whether these age-related changes were due to previous antigenic exposure, we performed a phenotypic and functional analysis on splenic CD4^+^ T cells isolated from individual, germ-free (GF), specific pathogen-free (SPF), and clean conventional (CC) mice. Interestingly, the total number of splenic CD4^+^ T cells in GF mice was twofold lower as compared to age-matched SPF or CC mice, regardless whether mice were analyzed at young (10 weeks) or at advanced age (13-14 months). Unexpectedly, the phenotypic composition of the CD4^+^ T-cell subset was comparable in the GF, SPF, and CC mice as determined by the expression of CD45RB and Pgp-1, indicating that CD4^+^ T cells with a naive phenotype (CD45RB^++^ Pgp-1 ^–^) were not enriched in GF mice. Moreover, at an age of 13–14 months, CD4^+^ T cells from GF mice frequently produced more IL-4 and IFN-*γ*, than their CC counterparts. These lymphokine data showed, therefore, that a relatively high proportion of CD4 T cells with a memory phenotype can also be defined in GF mice on the basis of their function. The contamination of GF mice with a colonization resistant factor (CRF flora) resulted in twofold higher numbers of splenic CD4^+^ T cells. Surprisingly, not only CD4^+^ T cells with a memory phenotype (CD45RB^–/+^ Pgp-1^++^) had expanded, but also CD4^+^ T cells with a naive (CD45RB^++^ Pgp-1^–^) phenotype. Our results, therefore, strongly suggest that the expansion of naive CD4^+^ T cells in the periphery is mediated by the intestinal microflora.


*Developmental Immunology*, 1992, Vol. 2, pp. 141–150
Reprints available directly from the publisher
Photocopying permitted by license only

© 1992 Harwood Academic Publishers GmbH
Printed in the United Kingdom

### The Involvement of the Intestinal Microflora in the Expansion of CD4$^+$ T Cells with a Naive Phenotype in the Periphery

RUUD DOBBER,† ANITA HERTOGH-HUIJBREGTS,† JAN ROZING,† KIM BOTTOMLY,‡ and LEX NAGELKERKEN*†

†*Section of Immunology, Institute of Ageing and Vascular Research TNO, P.O. Box 430, 2300 AK Leiden, The Netherlands*
‡*Section of Immunobiology, Howard Hughes Medical Institute, Yale University School of Medicine, New Haven, Connecticut*

It is well known that immune reactivity declines with age. Recently, we demonstrated that the age-related decrease in IL-2 production by CD4$^+$ T cells was accompanied by an increased production of IL-4 and interferon-$\gamma$ (IFN-$\gamma$). This age-related shift in the profile of lymphokine production was related to phenotypic changes within the CD4$^+$ T-cell subset, that is, a decrease in the percentage of CD45RB$^{++}$ CD4$^+$ T cells and an increase in the percentage of Pgp-1$^+$ CD4$^+$ T cells. To study whether these age-related changes were due to previous antigenic exposure, we performed a phenotypic and functional analysis on splenic CD4$^+$ T cells isolated from individual germ-free (GF), specific pathogen-free (SPF), and clean conventional (CC) mice. Interestingly, the total number of splenic CD4$^+$ T cells in GF mice was twofold lower as compared to age-matched SPF or CC mice, regardless whether mice were analyzed at young (10 weeks) or at advanced age (13–14 months). Unexpectedly, the phenotypic composition of the CD4$^+$ T-cell subset was comparable in the GF, SPF, and CC mice as determined by the expression of CD45RB and Pgp-1, indicating that CD4$^+$ T cells with a naive phenotype (CD45RB$^{++}$ Pgp-1$^-$) were not enriched in GF mice. Moreover, at an age of 13–14 months, CD4$^+$ T cells from GF mice frequently produced more IL-4 and IFN-$\gamma$ than their CC counterparts. These lymphokine data showed, therefore, that a relatively high proportion of CD4$^+$ T cells with a memory phenotype can also be defined in GF mice on the basis of their function.

The contamination of GF mice with a colonization resistant factor (CRF flora) resulted in twofold higher numbers of splenic CD4$^+$ T cells. Surprisingly, not only CD4$^+$ T cells with a memory phenotype (CD45RB$^{-/+}$ Pgp-1$^{++}$) had expanded, but also CD4$^+$ T cells with a naive (CD45RB$^{++}$ Pgp-1$^-$) phenotype. Our results, therefore, strongly suggest that the expansion of naive CD4$^+$ T cells in the periphery is mediated by the intestinal microflora.

KEYWORDS: CD4$^+$ T cells, germ-free, naive, memory, intestinal microflora.

### INTRODUCTION

Both phenotypic and functional changes occur within the CD4$^+$ T-cell subset during the aging process. It is well documented that CD4$^+$ T cells lose the capability to produce IL-2 with advanced age in response to specific antigen or polyclonal activators, for example, Con A (Gilman et al., 1982; Thoman and Weigle, 1982; Hertogh-Huijbregts et al., 1990). Recently, we and others demonstrated that this decline in IL-2 production is accompanied by an increased production of IL-4 and IFN-$\gamma$ (Ernst et al., 1990; Kubo and Cinader, 1990; Nagelkerken et al., 1991a). This shift in the profile of lymphokines produced by CD4$^+$ T cells appeared to be related to phenotypic changes within the CD4$^+$ T-cell subset, that is, an age-related decrease in the percentage of CD45RB$^{++}$ CD4$^+$ T cells and an increase in the percentage of Pgp-1$^+$ CD4$^+$ T cells (Lerner et al., 1989; Ernst et al., 1990; Nagelkerken et al., 1991a, 1991b).

Several investigators recently showed that the capability of CD4$^+$ T cells to respond to a "recall antigen" can be found within the CD45RB$^{low}$ CD4$^+$ T-cell population (Dianzani et al., 1990; Lee

*Corresponding author.

and Vitetta, 1990). Therefore, it is likely that the CD45RB$^{low}$ CD4$^+$ T cells reflect memory cells. Moreover, CD4$^+$ T cells that are distinct with regard to CD45RB expression produce different cytokines: CD45RB$^{high}$ CD4$^+$ T cells produce IL-2 and not IL-4, and CD45RB$^{low}$ CD4$^+$ T cells produce IL-4 and less IL-2 (Bottomly et al., 1989). It is therefore conceivable that IL-4 production is a characteristic of memory cells. Memory T cells can also be defined by the expression of Pgp-1. In mice, limiting dilution studies have shown that the Pgp-1$^+$ CD4$^+$ T-cell subset contains the antigen-specific T cells after immunization with keyhole limpet hemocyanin (Butterfield et al., 1989). Furthermore, Pgp-1$^+$ T cells are associated with the production of IFN-$\gamma$ (Budd et al., 1987). Altogether it seems likely that the age-related increase in the production of IL-4 and IFN-$\gamma$ by CD4$^+$ T cells reflects an increased number of memory CD4$^+$ T cells.

To study whether the observed age-related changes in the CD4$^+$ T-cell subset are due to previous antigenic exposure, we performed a phenotypic and functional analysis on splenic CD4$^+$ T cells isolated from individual mice with a different gnotobiotic background at young and advanced age. Our results show that the total number of splenic CD4$^+$ T cells isolated from SPF or CC mice is increased twofold as compared to GF mice. In spite of the lower numbers of CD4$^+$ T cells in GF mice, the composition of this population does not differ significantly from that in SPF and CC mice as far as phenotype and lymphokine production are concerned.

Contamination of GF mice with a CRF flora resulted not only in the expansion of CD4$^+$ T cells with a memory phenotype, but also in a comparable expansion of cells with a naive phenotype, indicating that the intestinal microflora play a pivotal role in the expansion and maintenance of naive CD4$^+$ T cells in the periphery.

### RESULTS

### Composition of Splenic Lymphocytes in GF, SPF, and CC Mice

Spleen cells from individual mice were isolated and subjected to FACS-analysis to determine the composition of spleens from mice with a different intestinal microflora. At an age of 10 weeks, the total number of CD3$^+$ T cells was twofold lower in GF mice as compared to SPF mice (Fig. 1, upper panel, $p<0.01$). In GF mice, both the numbers of CD4$^+$ T cells and CD8$^+$ T cells were significantly lower as compared to their SPF counterparts ($p<0.01$). Because the kinetics of the peripheral expansion of T cells in mice without an intestinal microflora may be slower, and in view of our interest in the contribution of previous antigenic exposure to age-related changes within the CD4$^+$ T-cell subset, we also analyzed 13-month-old GF and CC mice. As shown in Fig. 1 (lower panel), we still observed a significant difference ($p<0.01$) between the absolute number of T cells isolated from GF mice in comparison with CC mice. The absolute number of CD4$^+$ T cells in 13-month-old GF mice was two-fold lower than in CC mice ($p<0.01$). The absolute number of CD8$^+$ T cells in GF mice was not significantly decreased, while also no difference was found in the absolute number of B cells.

Lower numbers of CD4$^+$ T cells were also found in mesenteric lymph nodes and peripheral blood from GF mice regardless their age, indicating that this difference was not unique to the spleen (data not shown).

### Expression of CD45RB and Pgp-1 on Splenic CD4$^+$ T Lymphocytes from GF, SPF, and CC Mice

To evaluate the composition of the CD4$^+$ T-cell compartment in GF, SPF, and CC mice, we studied the expression of CD45RB and Pgp-1 on splenic CD4$^+$ T cells. This was done in double-staining experiments in which spleen cells were incubated with PE-labeled anti-L3T4 (anti-CD4) in conjunction with FITC-labeled anti-Pgp-1 or in the combination of anti-CD45RB and FITC-labeled mouse–antirat Ig (for details, see Materials and Methods). The expression of CD45RB or Pgp-1 was determined on gated CD4$^+$ T cells. Our marker setting of CD45RB and Pgp-1 was based on the expression of these molecules on splenic CD4$^+$ T cells from 1-year-old mice. As shown in Fig. 2(A), at least three populations can be defined in 1-year-old mice based on the expression of CD45RB: CD45RB$^-$ CD4$^+$, CD45RB$^+$ CD4$^+$, and CD45RB$^{++}$ CD4$^+$. Similarly, at least three populations can be discriminated on the basis of Pgp-1 expression, Fig. 2(B): Pgp-1$^-$ CD4$^+$, Pgp-1$^+$ CD4$^+$, and Pgp-1$^{++}$ CD4$^+$. Contour plots of double-staining experiments on enriched CD4+ T cells revealed that within the CD45RB++ population in GF, Figs. 3(B) and 3(D), and CC or SPF, Figs. 3(A) and 3(C), at young and advanced age, at least two expression levels of Pgp-1 exist: CD45RB++ Pgp-1- and CD45RB++ Pgp-1+. In addition, in 1-year-old CC mice as well as in 1-year-old GF mice, a CD45RB+ Pgp-1++ CD4+ T-cell population could be recognized. The CD45RB- CD4+ T-cell population, evident from double-staining experiments in the total spleen-cell populations, was not observed in the analysis of the purified CD4+ T cells. This is possibly due to the detection of rat antibodies, aspecifically bound to the CD4+ T cells during the isolation procedure, resulting in a higher background staining with FITC-labeled mouse–antirat Ig. These contour plots demonstrate that at an age of 10 weeks, little CD4+ T cells with a memory phenotype, that is, CD45RB-/+ Pgp-1++, can be detected; see Figs. 3(C) and 3(D). At an age of 13–14 months, however, this CD4+ T-cell popu-

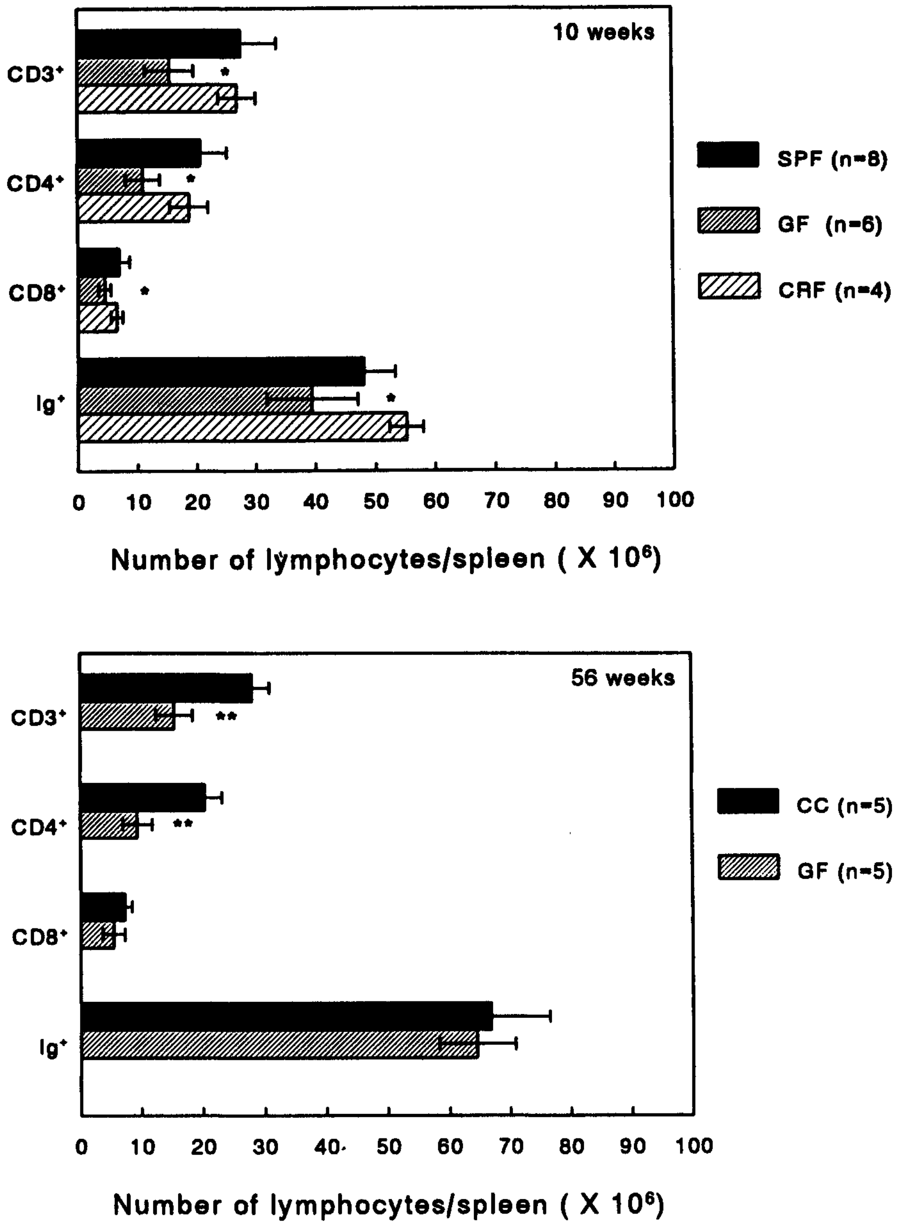

FIGURE 1. Influence of the intestinal microflora on the splenic lymphocyte composition. Absolute numbers of splenic lymphocytes were determined by FACS analysis in male CBA/Rij mice with a different intestinal microflora at an age of 10 weeks (uper panel) or 56 weeks (lower panel). n=number of individual mice analyzed per group; error bars indicate standard deviations. *$p<0.01$ as compared to their SPF counterparts. **$P<0.01$ as compared to their CC counterparts.

lation was clearly present, irrespective whether it concerned CC mice, Fig. 3(A), or GF mice Fig. 3(B).

As reported earlier, antigenic exposure may influence the development of CD45RB$^{low}$ CD4$^{+}$ T cells (Dianzani et al., 1990; Lee et al., 1990; Lee and Vitetta, 1990). We therefore had expected that—in analogy with the findings of Lee et al. (1990)—the relative number of CD4$^{+}$ T cells with a naive phenotype would have been much higher in GF mice as compared to SPF or CC mice. However, as can be deduced from the contour plots, the distribution of CD45RB and Pgp-1 on splenic CD4$^{+}$ T cells was comparable between GF mice and SPF or CC mice. Furthermore this is illustrated by the absolute numbers of the different CD4$^{+}$ T-cell populations, as determined by FACS analysis. To determine the percentages of the different CD4$^{+}$ T-cell populations in young mice, we used the marker setting as indicated in Figs 2(A) and 2(B). As shown in Table 1 and Table 2, the lower number of CD4$^{+}$ T cells in GF mice as compared to SPF or CC mice could not exclusively be attributed to the lack of expansion of cells with a memory phenotype. Also the absolute number of naive CD4$^{+}$ T cells (i.e., CD45RB$^{++}$ in Table 1 or Pgp-1$^{-}$ in Table 2) was twofold lower. For example, at an age of 10 weeks—on the basis of these markers—the number of naive CD4$^{+}$ T cells per spleen was 6 to 7×10$^{6}$ in GF mice and 12 to 14 ×10$^{6}$ in SPF mice.

These data therefore suggest that the intestinal microflora has an influence on the peripheral expansion of memory CD4$^{+}$ T cells as well as naive CD4$^{+}$ T cells.

### GF Mice Contaminated with a Colonization Resistant Factor Flora Are Comparable with SPF Mice

To establish directly the effect of the intestinal microflora on both the number and phenotype of splenic lymphocytes, GF mice were contaminated at an age of 3 weeks with a colonization resistant factor flora (CRF-flora) (van Bekkum et al., 1974). This CRF-flora is an anaerobic microflora largely composed of spore forming gram-positive rods (Wensinck and Ruseler-van-Embden, 1971). Seven weeks after the contamination, the number and composition of the CD4$^{+}$ T cells in these CRF mice were determined.

As shown in Fig. 1, the total number of CD4$^{+}$ T cells in CRF mice was comparable to that in SPF mice (i.e., about 20×10$^{6}$ cells/spleen) and hence twofold higher than that in uncontaminated GF mice.

Analysis of CD45RB and Pgp-1 expression on CD4$^{+}$ T cells of these CRF mice revealed that also

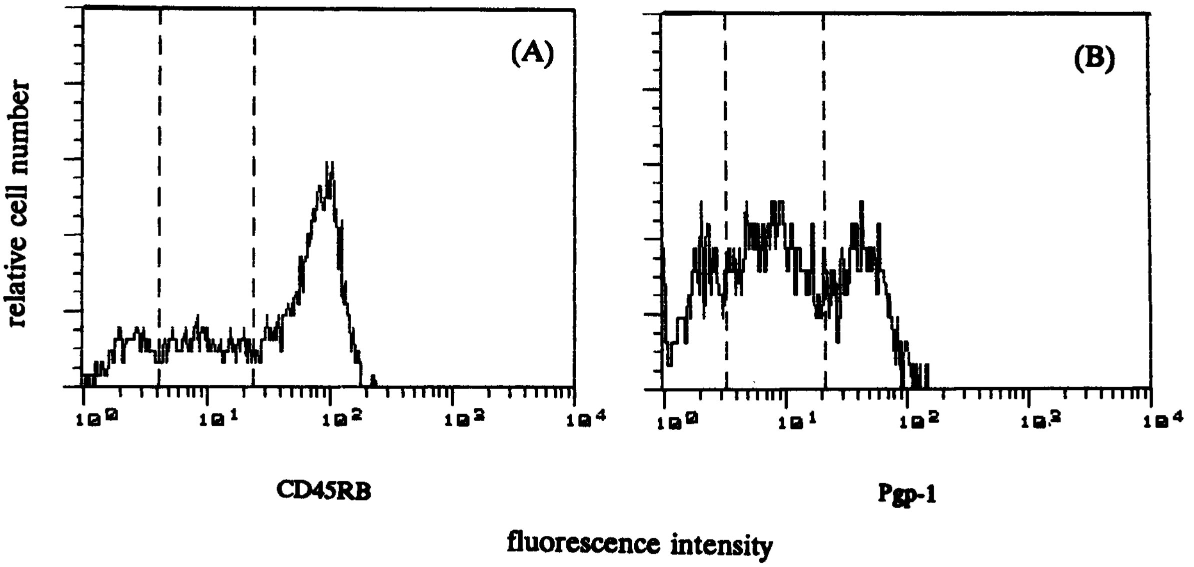

FIGURE 2. Expression of CD45RB (A) and Pgp-1 (B) on splenic CD4$^{+}$ T cells from a 1-year-old GF mouse. Splenocytes were incubated with mAb 16A and FITC-labeled mouse–antirat-Ig in combination with PE-labeled anti-CD4. Alternatively, cells were incubated with FITC-labeled anti-Pgp-1 in combination with PE-labeled anti-CD4. Histograms are displayed for gated CD4$^{+}$ T cells.

the expression patterns of these molecules were comparable with that in SPF and GF mice (Tables 1 and 2). Therefore, these data clearly demonstrate, the contamination of GF mice with a CRF flora resulted not only in the expansion of $CD4^+$ T cells with a memory phenotype, but also of $CD4^+$ T cells with a naive phenotype.

### Lymphokine Production by $CD4^+$ T Cells Isolated from GF, SPF, and CC Mice

Apart from a distinction between $CD4^+$ T-cell subsets on the basis of phenotype, a discrimination can also be made on the basis of the lymphokine production by $CD4^+$ T cells. We therefore studied the production of IL-2, IL-4, and IFN-$\gamma$ by purified $CD4^+$ T cells from individual GF, SPF, and CC mice after stimulation with Con A in the presence of irradiated accessory cells. Three representative experiments are depicted in Table 3. IL-2 production by $CD4^+$ T cells was comparable between 10-week-old GF or SPF mice. In agreement with previous studies, the amounts of IL-4 and IFN-$\gamma$ were below the detection limit (Powers et al., 1988; Ernst et al., 1990; Nagelkerken et al., 1991a). $CD4^+$ T cells derived from 13–14-month-old mice produced lower levels of IL-2 as compared to 10-week-old mice. Again, no differences between GF and CC mice were noted. In contrast to the observations made in young mice, IL-4 and IFN-$\gamma$ could now easily be detected in CC mice as well as in GF mice. The fact that $CD4^+$ T cells from GF mice produce relatively high amounts of IL-4 and IFN-$\gamma$ was unexpected based on the assumption that these mice would have only naive $CD4^+$ T cells, but is in line with our phenotypic observations as described before.

### DISCUSSION

Previous studies have indicated that with advanced age, the maturation of the $CD4^+$ T-cell

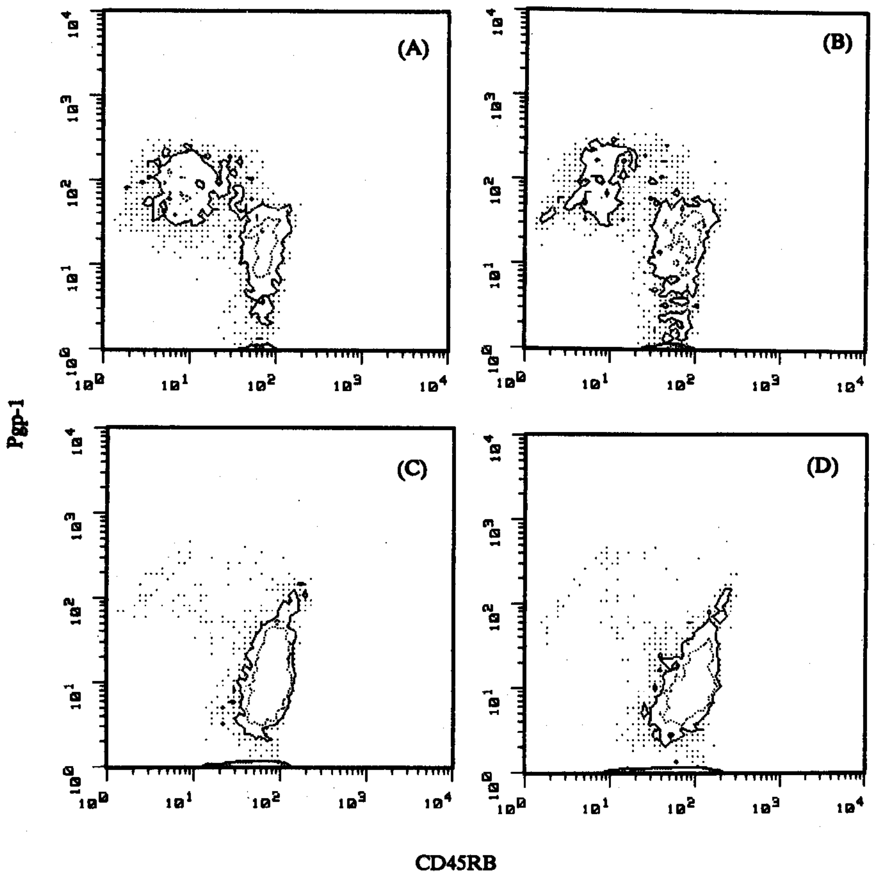

FIGURE 3. Coexpression of CD45RB and Pgp-1 on splenic $CD4^+$ T cells from a 1-year-old CC mouse (A), a 1-year-old GF mouse (B), a 10-week-old SPF mouse (C), and a 10-week-old GF mouse (D). Splenic $CD4^+$ T cells were isolated as described in Materials and Methods, and incubated with mAb 16A and FITC-labeled mouse–antirat Ig in combination with PE-labeled anti-Pgp-1. Contour plots of $CD4^+$ T cells were generated from 16,000 events.

TABLE 1
Influence of the Intestinal Microflora on the Expression of CD45RB on CD4+ T Cells[a]

| | CD45RB− | CD45RB+ | CD45RB++ |
|---|---|---|---|
| Young | | | |
| SPF (n=8) | 2.6±0.8[b] | 5.9±2.0 | 12.2±2.8 |
| GF (n=6) | 1.5±0.4 | 3.6±1.2 | 5.9±1.8 |
| CRF (n=4) | 2.3±0.2 | 6.3±0.4 | 10.2±3.5 |
| Aged | | | |
| CC (n=5) | 3.4±1.4 | 5.4±0.9 | 11.5±0.8 |
| GF (n=5) | 2.1±0.9 | 2.2±0.5 | 5.0±1.7 |

[a]Unseparated spleen cells were incubated with mAb 16A and FITC-labeled mouse–antirat Ig in combination with PE-labeled anti-CD4. For analysis of CD45RB expression on CD4+ T cells, only gated PE-positive cells were included.
[b]Results represent the mean±SD of the absolute numbers of the different CD4+ T-cell subsets of mice with a different intestinal microflora at young (10 weeks) and at advanced age (56 weeks). The marker setting was used as indicated in Fig. 1. n= number of individual mice analyzed per group.

TABLE 2
Influence of the Intestinal Microflora on the Expression of Pgp-1 on CD4+ T Cells[a]

| | Pgp-1− | Pgp-1+ | Pgp-1++ |
|---|---|---|---|
| Young | | | |
| SPF (n=8) | 14.7±4.3[b] | 4.2±0.8 | 1.9±0.3 |
| GF (n=6) | 7.7±2.7 | 2.0±1.0 | 1.3±0.2 |
| CRF (n=4) | 14.3±2.7 | 3.1±0.4 | 1.3±0.2 |
| Aged | | | |
| CC (n=5) | 5.6±2.9 | 10.1±1.1 | 4.7±1.5 |
| GF (n=5) | 2.8±2.0 | 4.3±1.1 | 2.2±0.8 |

[a]Unseparated spleen cells were incubated with FITC-labeled Pgp-1 in combination with PE-labeled-anti-CD4. For analysis of Pgp-1 expression on CD4+ T cells, only gated PE-positive cells were included.
[b]Results represent the mean±SD of the absolute numbers of the different Pgp-1 CD4+ T-cell subsets of male mice with a different intestinal microflora at young (10 weeks) and advanced age (56 weeks). The marker setting used is indicated in Fig. 1. n=number of individual mice analyzed per group.

TABLE 3
Lymphokine Production by Con A-Stimulated CD4+ T Cells Isolated from Mice with a Different Intestinal Microflora at Young and Advanced Age[a]

| | IL-2[b] (U/mL) | IL-4[b] (U/mL) | IFN-γ[b] (ng/mL) |
|---|---|---|---|
| Expt. 1: young[c] ♂ | | | |
| SPF-1 | 67 | <1[d] | <1 |
| SPF-2 | 67 | <1 | <1 |
| GF-1 | 64 | <1 | <1 |
| GF-2 | 62 | <1 | <1 |
| Expt. 2: aged[e] ♂ | | | |
| CC-3 | 28 | 2 | 10 |
| CC-4 | 30 | 3 | 3 |
| CC-5 | 19 | 6 | 23 |
| GF-3 | 5 | 17 | 20 |
| GF-4 | 29 | 1 | 12 |
| GF-5 | 16 | 8 | 6 |
| Expt. 3: aged[f] ♀ | | | |
| CC-6 | 32 | 2 | 9 |
| CC-7 | 26 | 3 | 4 |
| CC-8 | 54 | <1 | 2 |
| GF-6 | 14 | 11 | 5 |
| GF-7 | 45 | 16 | 23 |
| GF-8 | 38 | 52 | 54 |

[a]CD4+ T cells were isolated from individual SPF, GF, or CC mice and cultured as described in Materials and Methods. .

[b]IL-2, IL-4, and IFN-γ concentrations in supernatants harvested at day 3.

[c]10-week-old mice.

[d]<1: less than 1 U IL-4/mL or 1 ng IFN-γ/mL.

[e]56-week-old mice.

[f]60-week-old mice.

compartment is reflected by both phenotypic and functional changes. Phenotypically, this was shown by a decreased expression of CD45RB and an increased expression of Pgp-1 on the CD4+ T cells, and the functional changes were reflected by the decrease in IL-2 production and the increase in IL-4 and IFN-γ production by CD4+ T cells (Lerner et al., 1989; Kubo and Cinader, 1990; Ernst et al., 1990; Nagelkerken et al., 1991a). Therefore, IL-4 and IFN-γ can be considered as lymphokine products of previously activated CD4+ T cells. Evidence is accumulating that previous antigenic exposure may influence the composition of the CD4+ T-cell subset: After immunization, the antigen-specific CD4+ T cells can be found within the Pgp-1+ CD4+ T-cell population (Butterfield et al., 1989) and/or the CD45RB$^{low}$ CD4+ T-cell population (Dianzani et al., 1990; Lee and Vitetta, 1990). It is likely, therefore, that the accumulation of CD45RB$^{low}$ Pgp-1+ CD4+ T cells in old mice is due to previous antigenic exposure.

In order to obtain further proof for this possibility, CD4+ T cells from mice with a different intestinal microflora, that is, germ-free (GF) and specific pathogen-free (SPF) or clean conventional mice (CC) were studied with regard to phenotype and function. We showed that the absolute number of splenic CD4+ T cells in male GF mice was twofold lower as compared to SPF or CC mice, regardless whether it concerned 10-week-old or 13-month-old mice (Fig. 1). Our findings in young mice are consistent with those described recently by Bandeira et al. (1990). They showed that the intestinal microbial colonization has little effect on either the numbers or the phenotype of T-cell receptor γδ expressing intraepithelial lymphocytes in GF and SPF mice. However, they also observed significantly lower numbers of T-cell receptor *αb* expressing intraepithelial lymphocytes and splenic T cells in GF mice when compared with SPF mice.

To study whether the observed differences between GF mice and SPF mice are indeed due to the intestinal microflora, we exposed GF mice to a CRF flora (an anaerobic microflora that prevents the colonization of pathogenic microorganisms) at an age of 3 weeks. Analysis of these CRF mice at an age of 10 weeks showed that the total number of splenic $CD4^+$ T cells was comparable to the total number of splenic $CD4^+$ T cells in SPF mice and thus twofold higher than that in uncontaminated GF mice.

Relatively little is known about the composition of $CD4^+$ T cells in GF mice. We therefore studied the expression of CD45RB and Pgp-1 on these cells. If antigenic exposure (e.g., an intestinal microflora) influences the composition of the $CD4^+$ T-cell population, one would expect that in GF mice, the contribution of $CD4^+$ T cells with a naive phenotype (i.e., $CD45RB^{++}$ $Pgp\text{-}1^-$) would have been much higher in comparison with SPF or CC mice. Surprisingly, the relative contributions of the naive and memory $CD4^+$ T cell subsets in GF and SPF or CC mice were identical. So, the lower number of $CD4^+$ T cells in GF mice could not exclusively be attributed to the absence of a specific $CD4^+$ T-cell subset, for example, memory cells. Moreover, also the contamination of GF mice with a CRF flora did not result in a preferential expansion of memory $CD4^+$ T cells.

Our results are in contrast to the findings of Lee et al. (1990) who demonstrated that the majority of $CD4^+$ T cells in GF BALB/c mice has a naive phenotype, whereas a dramatic increase of cells with a memory phenotype occurs after antigenic exposure. First of all, we observed higher numbers of memory $CD4^+$ T cells not only in the GF CBA/Rij mice we used in this study, but also in GF BALB/c mice (data not shown). We attribute the presence of these cells to the exposure of the mice to food antigens. In addition, the fact that we studied the influence of an intestinal microflora rather than immunizing with keyhold limpet hemocyanin or alloantigens may be the reason that we do not observe a preferential expansion of memory $CD4^+$ T cells in the periphery.

It might be argued that the kinetics of the expansion of $CD4^+$ T cells in GF mice is slower. However, we observed that at an age of 13 months, the absolute number of $CD4^+$ T cells in GF mice was still twofold lower. The presence of memory $CD4^+$ T cells in GF mice was further confirmed by the observation that 13–14-month-old GF mice were not deficient in IL-4 and IFN-$\gamma$ producing $CD4^+$ T cells.

In conclusion, this study indicates that in GF mice—as compared to SPF mice—not only the number of memory $CD4^+$ T cells is low, but also that of naive $CD4^+$ T cells. Therefore, it can be concluded that in SPF or CC mice, the presence of an intestinal microflora has equally stimulated the expansion of naive $CD4^+$ T cells and of memory $CD4^+$ T cells in the periphery as was also confirmed by introducing a CRF flora in GF mice.

It might be that the intestinal microflora affects the output of the thymus. Alternatively, it might be that antigens associated with the microflora are responsible for the expansion or a prolonged self-renewal capacity of naive $CD4^+$ T cells in the periphery in SPF or CC mice. In early studies by Kappler and colleagues, it has been shown that the life-span of naive $CD4^+$ T cells is about 10 weeks. However, in this study, a long-lived naive cell population (15% of the total T-cell population) remained present in these thymectomized mice (Kappler et al., 1974). Further evidence for the ability of $CD4^+$ T cells to expand in the periphery has been presented by Bell et al. (1987) in nude rats. In addition, Rocha and colleagues showed that peripheral T cells are able to self-renew and to expand after transfer into T cell-deficient mice, without the requirement of exogenous stimulation (Rocha et al., 1989). Without referring to the microbial status of the recipients, the authors suggest that "homeostatic mechanisms" influence the expansion of T cells in the periphery. On the basis of our study, it is likely that the presence of an intestinal microflora in SPF or CC mice results in a different homeostatic regulation of peripheral T cells as compared to GF mice. It is therefore tempting to hypothesize that microorganisms or their products (e.g., superantigens) are involved in the maintenance and expansion of $CD4^+$ T cells in the periphery. This possibility is in line with studies by De Geus and colleagues, who showed that a high degree of variability in T-cell receptor usage is found in intraepithelial lymphocytes and that this may be a reflection of individual variability of the intestinal flora between the different mice (De Geus et al., 1990).

### MATERIALS AND METHODS

### Mice

Female- and male-specific pathogen-free (SPF), colonization resistent factor (CRF), and germ-free (GF) CBA/Rij mice of different ages were obtained from the animal facilities from the Institute for Applied Radiobiology and Immunology TNO, Rijswijk, The Netherlands. Aged mice were obtained from the SPF stock and housed under clean conventional conditions behind a physical barrier from the age of 3 months. These mice will further be referred to as clean conventional mice (CC).

### Phenotypic Analysis

For the enumeration of T cells, we used FITC-labeled anti-CD3 (mAb 145-2C11 (Leo et al., 1987), which was kindly provided by Dr. J. A. Bluestone). $CD4^+$ T cells were detected either by using FITC-labeled anti-L3T4, produced by clone GK1.5 (Dialynas et al., 1983) or by using PE-labeled anti-L3T4 (Becton Dickinson, Sunnyvale, CA). For the detection of $CD8^+$ T cells, we used FITC-labeled anti-lyt2 (Becton Dickinson). Alternatively, $CD8^+$ T cells were determined using biotin-labeled anti-lyt2 (Becton Dickinson) in combination with PE-streptavidin (Becton Dickinson). B cells were detected with the use of FITC-labeled goat–antimouse Ig (Nordic, Tilburg, The Netherlands). Pgp-1 (CD44) expression was studied using FITC- or PE-labeled anti-Pgp-1 (Pharmingen, San Diego, CA). CD45RB expression was examined with the use of mAb 16A (Bottomly et al., 1989). As a second antibody, we used FITC-conjugated mouse–antirat Ig antibody (Jackson Immunoresearch Lab., West Grove, PA). Phenotypic analysis was done by incubating $0.5\times10^6$ cells, 30 min on ice, with an appropriate dilution of one of these mAbs. For the double-staining experiments, cells were first incubated with mAb 16A and FITC-labeled mouse–antirat Ig. After washing the cells, the second incubation step consisted of PE-labeled anti-L3T4 or PE-labeled Pgp-1 in the presence of 10% rat serum to avoid binding to the second developing antibody from the first step. After washing, the cells were analyzed with the use of a FACScan (Becton Dickinson).

### Isolation of $CD4^+$ T Cells

Spleens from individual mice were aseptically removed. Single-cell suspensions were prepared in RPMI 1640 (Gibco, Paisley, Scotland) supplemented with penicillin (100 IU/mL), streptomycin (100 $\mu$g/mL), L-glutamine (2 g/L). $\beta$-mercaptoethanol ($5\times10^{-5}$ M), HEPES (20 mM), and 5% fetal calf serum (FCS, Seralab, Crawley Down, UK). The total number of spleen cells was assessed prior to lysis of the erythrocytes with ammoniumchloride, after which the cells were centrifuged over a FCS layer (20 min, 50×g, 4 °C). Cells were resuspended in medium and incubated for 30 min on ice with a cocktail of the antibodies anti-lyt2 (55.6.7), anti-Mac1$\alpha$ (American Type Culture Collection, ATCC, Rockville, MD; TIB 128), and anti-I-$A^{b,d,q}$ (ATCC; TIB 120) in order to deplete $CD8^+$ T cells and the majority of macrophages and B cells. Thereafter, cells were washed to remove unbound mAb and incubated ($10^8$ cells/mL, room temperature) with 25% Magnisort–antimouse suspension as well as 25% goat–antirat labeled Magnisort–antigoat suspension, according to the instructions of the manufacturer (DuPont, Wilmington, DE). The adherent fraction was removed with a Dynal MPC-6 Magnetic Particle Concentrator (Dynal AS, Oslo, Norway). With this method, splenic $CD4^+$ T cells were enriched to about 90%.

### Cell Cultures

Fifty thousand enriched $CD4^+$ T cells from individual mice were cultured in 96-well flat-bottomed microtiter plates (Costar, Cambridge, UK) in medium as described before. Cells were stimulated with an optimal concentration of Con A (1 $\mu$g/mL, Pharmacia) in the presence of $10^5$ 2500 rad-irradiated syngeneic young spleen cells, which served as a source of accessory cells. Supernatants were collected after several culture periods and stored frozen at −20 °C until assay.

### Cytokine Assays

IL-2 was assayed with the use of CTLL-2 cells. Five thousand cells were cultured in the presence of serially diluted supernatants with a neutralizing amount anti-IL-4 (5 $\mu$g/mL 11.B.11; Ohara and Paul, 1985). During the last 4 hr of the culture period of 24 hr, cells were pulsed with 0.25

μCi [$^3$H]dThd (Radiochemical Center, Amersham, UK). IL-4 was assayed with the use of the IL-4-dependent CT.4S cell line (kindly provided by Dr. G. Legros, Ciba Geigy Ltd., Basel, Switzerland; Hu-Li et al., 1989). Again, 5×10$^3$ cells were cultured in the presence of serially diluted supernatants. Cells were pulsed with 0.25 μCi [$^3$H]dThd from 40–48 hr of culture. Supernatants of transfectants secreting recombinant murine IL-2 or IL-4 (Karasuyama and Melchers, 1988) served as a standard to prepare the relevant calibration curves (kindly provided by Dr. F. Melchers, Basel Institute for Immunology, Basel, Switzerland). One U/mL is defined as the concentration that results in the half-maximal proliferation of the CTLL-2 or CT.4S cells. IFN-γ was assayed by ELISA (Holland Biotechnology, Leiden, The Netherlands).

### Statistical Analysis

Results were subjected to statistical analysis by using the Wilcoxon test.

### ACKNOWLEDGMENTS

The authors would like to thank Mr. Tom Glaudemans for providing the photographs and Drs. Eric Braakman and Paula van den Bergh for critically reviewing the manuscript.

*(Received May 14, 1991)*

*(Accepted September 25, 1991)*

### REFERENCES

Bandeira A., Mota-Santos T., Itohara S., Degermann S., Heusser C., Tonegawa S., and Couthinho A. (1990). Localization of γ/δ cells to the intestinal epithelium is independent of normal microbial colonization. J. Exp. Med. **172:** 239–244.

Bell E.B., Sparshott S.M., Drayson M.T., and Ford W.L. (1987). The stable and permanent expansion of functional T lymphocytes in athymic nude rats after a single injection of mature T cells. J. Immunol. **139:** 1379–1384.

Bottomly K., Luqman M., Greenbaum L., Carding S., West J., Pasqualini T., and Murphy D.B. (1989). A monoclonal antibody to murine CD45R distinguishes CD4 T cell populations that produce different cytokines. Eur. J. Immunol. **19:** 617–623.

Budd R.C., Cerottini J.-C., and McDonald H.R. (1987). Selectively increased production of interferon-γ by subsets of lyt-2+ and L3T4+ T cells identified by expression of Pgp-1. J. Immunol. **138:** 3583–3587.

Butterfield K., Fathman C.G., and Budd R.C. (1989). A subset of memory CD4+ helper T lymphocytes identified by expression of Pgp-1. J. Exp. Med. **169:** 1461–1466.

De Geus B., Van den Enden-Vieveen M.H.M., De Heer C., Kleiweg A., Heidt P., Timmermans C., and Rozing J. (1990). The intestinal intraepithelial lymphocyte population expressing the TCR *αb* shows variation in Vβ usage between individual mice. *In:* From gene to man, Bezooijen C.F.A., Ravid R., and Verhofstad A.A.J., eds. ('s-Gravenhage, The Netherlands: Pasmans), pp. 313–315.

Dialynas D.P., Wilde D.B., Marrack P., Pierres A., Wall K.A., Havran W., Otten G., Loken M.R., Pierres M., Kappler J., and Fitch F.W. (1983). Characterization of the murine antigenic determinant, designated L3T4a, recognized by monoclonal antibody GK1.5: Expression of L3T4a by functional T cell clones appears to correlate primarily with class II MHC antigen-reactivity. Immunol. Rev. **74:** 29–56.

Dianzani U., Luqman M., Rojo J., Yagi J., Baron J.L., Janeway Jr., C.A., and Bottomly K. (1990). Molecular association on the T cell surface correlate with immunological memory. Eur. J. Immunol. **20:** 2249–2257.

Ernst D.N., Hobbs M.V., Torbett B.E., Glasebrook A.L., Rehse M.A., Bottomly K., Hayakawa K., Hardy R.R., and Weigle W.O. (1990). Differences in the expression profiles of CD45RB, Pgp-1, and 3G11 membrane antigens and in the patterns of lymphokine secretion by splenic CD4+ T cells from young and aged mice. J. Immunol. **145:** 1295–1302.

Gilman S.C., Rosenberg J.S., and Feldman J.D. (1982). T lymphocytes of young and aged rats. II. Functional defects and the role of interleukin-2. J. Immunol. **128:** 644–650.

Hertogh-Huijbregts A., Vissinga C., Rozing J., and Nagelkerken L. (1990). Impairment of CD3-dependent and CD3-independent activation pathways in CD4+ and CD8+ T cells from old CBA/Rij mice. Mech. Aging Dev. **53:** 141–155.

Hu-Li J., Ohara J., Watson C., Tsang W., and Paul W.E. (1989). Derivation of a T cell line that is highly responsive to IL-4 and IL-2 (CT.4R) and of an IL-2 hyporesponsive mutant of that line (CT.4S). J. Immunol. **142:** 800–807.

Kappler J.W., Hunter P.C., Jacobs D., and Lord E. (1974). Functional heterogenity among the T-derived lymphocytes of the mouse. J. Immunol. **113:** 27–38.

Karasuyama H., and Melchers F. (1988). Establishment of mouse cell lines which constitutively secrete large quantities of interleukin 2, 3, 4 or 5 using modified cDNA expression vectors. Eur. J. Immunol. **18:** 97–104.

Kubo M., and Cinader B. (1990). Polymorphism of age-related changes in interleukin (IL) production: Differential changes of T helper subpopulations, synthesizing IL 2, IL 3 and IL 4. Eur. J. Immunol. **20:** 1289–1296.

Lee W.T., and Vitetta E.S. (1990). Limiting dilution analysis of CD45R$^{hi}$ and CD45R$^{lo}$ T cells: Further evidence that CD45R$^{lo}$ cells are memory cells. Cell. Immunol. **130:** 459–471.

Lee W.T., Yin X.-M., and Vitteta E.S. (1990). Functional and ontogenetic analysis of murine CD45R$^{hi}$ and CD45R$^{lo}$ CD4+ T cells. J. Immunol. **144:** 3288–3295.

Leo O., Foo M., Sachs D.H., Samelson L.E., and Bluestone J.A. (1987). Identification of a monoclonal antibody specific for a murine T3 polypeptide. Proc. Natl. Acad. Sci. USA **84:** 1374–1378.

Lerner A., Yamada T., and Miller R.A. (1989). Pgp-1$^{hi}$ T lymphocytes accumulate with age in mice and respond poorly to concanavalin A. Eur. J. Immunol. **19:** 977–982.

Nagelkerken L., Hertogh-Huijbregts A., Dobber R., and Dräger A. (1991a). Age-related changes in lymphokine production related to a decreased number of CD45RB$^{hi}$ CD4+ T cells. Eur. J. Immunol. **21:** 273–281.

Nagelkerken L., Hertogh-Huijbregts A., and Dobber R. (1991b). Influence of maturation on T-cell reactivity. *In:* Lymphatic tissues in vivo immune responses, Imhof B., Ed. (New York: Marcel Dekker), pp. 227–231.

Ohara J., and Paul W.E. (1985). Production of a monoclonal antibody to and molecular characterization of B-cell stimulatory factor-1. Nature **315:** 333–336.

Powers G.D., Abbas A.K., and Miller R. (1988). Frequencies of IL-2- and IL-4-secreting T cells in naive and antigen-stimulated lymphocyte populations. J. Immunol. **140:** 3352–3357.

Rocha B., Dautigny N., and Pereira P. (1989). Peripheral T lymphocytes: Expansion potential and homeostatic regulation of pool sizes and CD4/CD8 ratios in vivo. Eur. J. Immunol. **19:** 905–911.

Thoman M.L., and Weigle W.O. (1982). Cell-mediated immunity in aged mice: An underlying lesion in IL-2 synthesis. J. Immunol. **128:** 2358–2361.

Van Bekkum D.W., Roodenburg J., Heidt P.J., and Van der Waaij D. (1974). Mitigation of secondary disease of allogeneic mouse radiation chimeras by modification of the intestinal microflora. J. Natl. Cancer Inst. **52:** 401–404.

Wensinck F., and Ruseler-van Embden J.G.H. (1971). The intestinal flora of colonization resistant mice. J. Hyg. Camb. **69:** 413–420.

